# Supplementary material for: Seminoma and Embryonal Carcinoma Footprints Identified by Analysis of Integrated Genome-Wide Epigenetic and Expression Profiles of Germ Cell Cancer Cell Lines
Source: PLoS One. 2014 Jun 2;9(6):e98330. doi: 10.1371/journal.pone.0098330 (PMC4041891; doi:10.1371/journal.pone.0098330)
Supplement: File S1 — ZIP file containing DMRforPairs output for significant regions. Please start from the html files. (ZIP) [file pone.0098330.s008.zip › figures/40410.pdf]

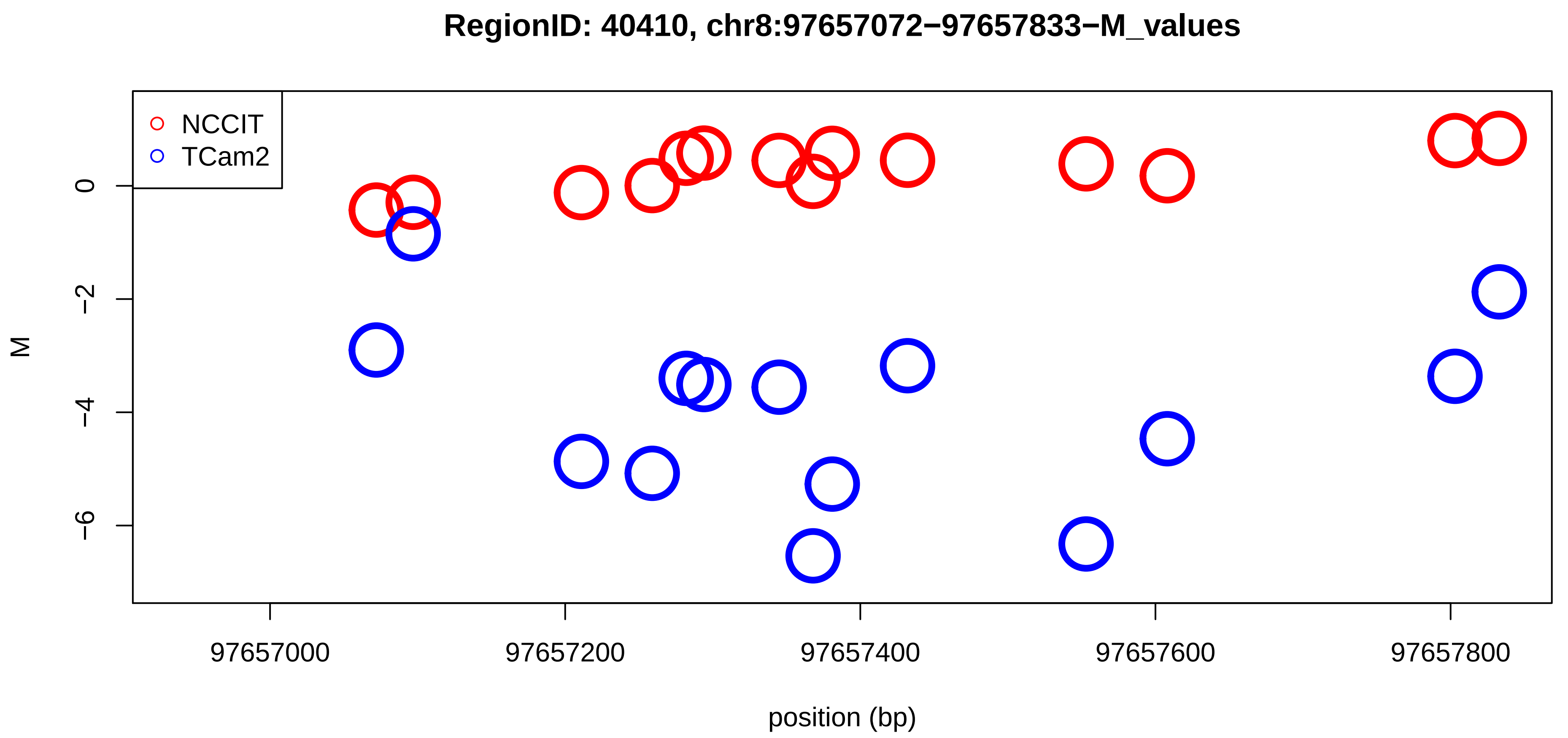
RegionID: 40410, chr8:97657072−97657833−M_values
NCCIT
TCam2
M
0
−2
−4
−6
97657000
97657200
97657400
97657600
97657800
position (bp)

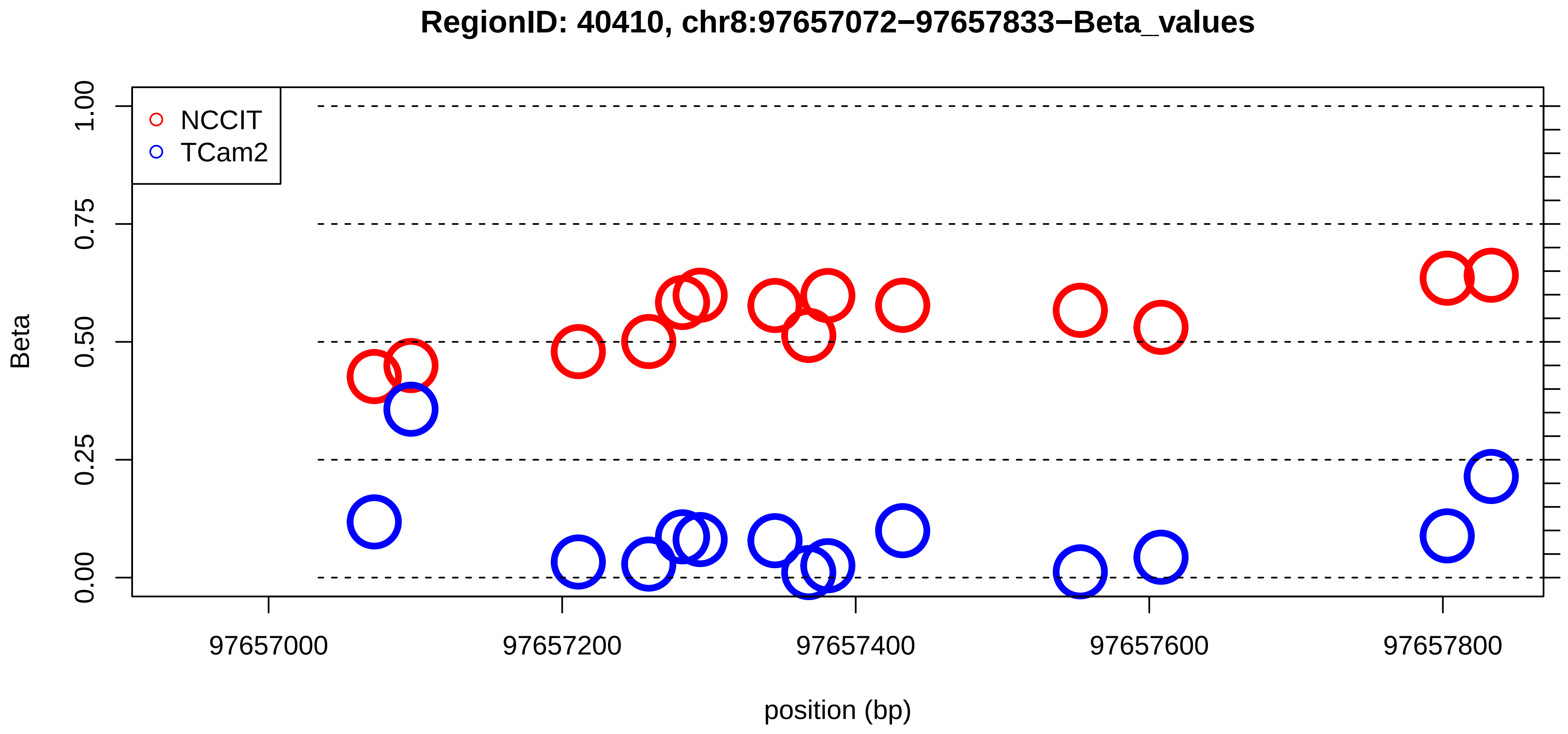
RegionID: 40410, chr8:97657072−97657833−Beta_values
NCCIT
TCam2
Beta
0.00
0.25
0.50
0.75
1.00
97657000
97657200
97657400
97657600
97657800
position (bp)

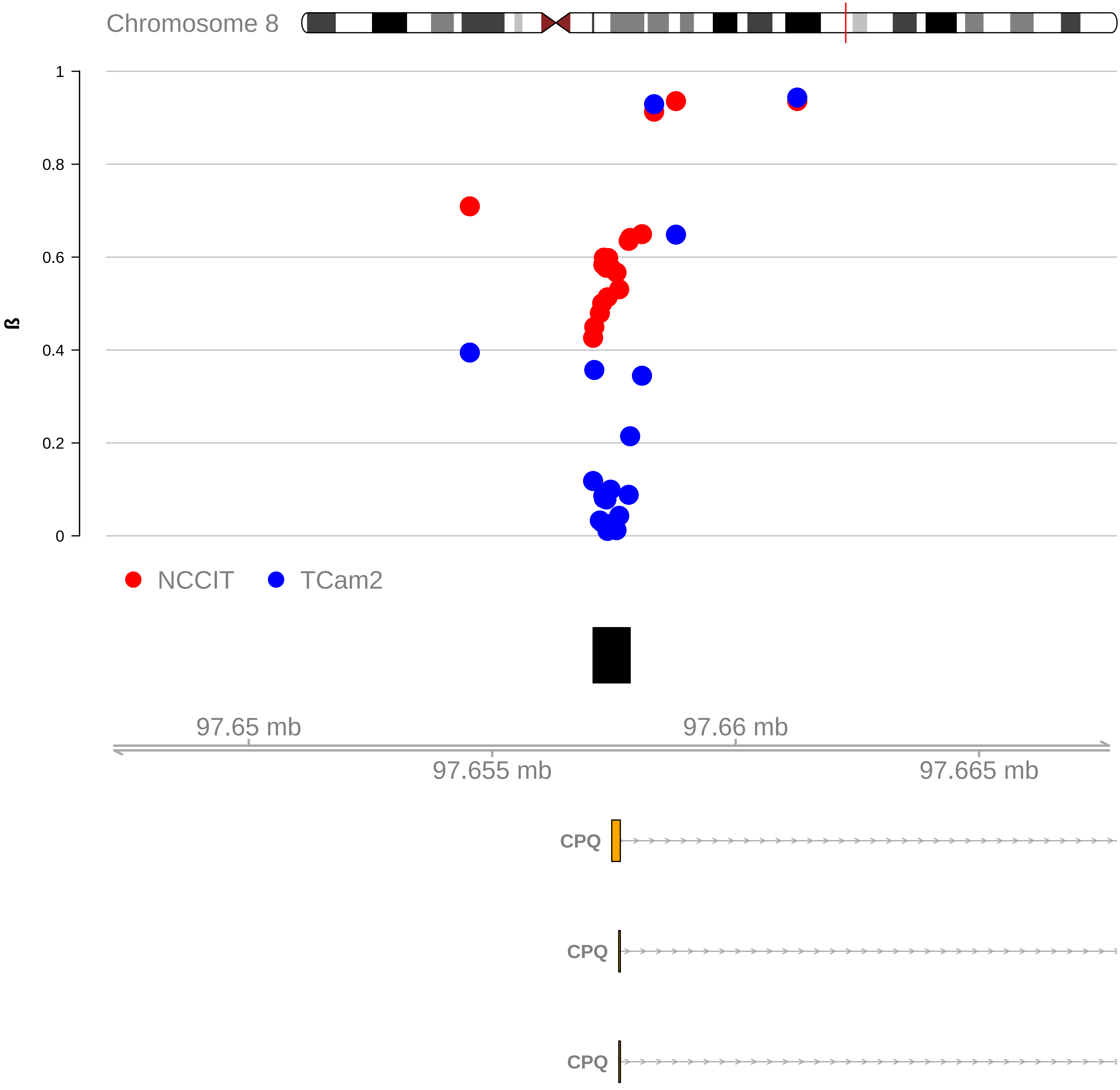

Chromosome 8
1
0.8
0.6
0.4
0.2
0
ß
NCCIT
TCam2
97.65 mb
97.66 mb
97.655 mb
97.665 mb
CPQ
CPQ
CPQ
